# Patients’ experiences of alcohol screening and advice in primary care: a qualitative study

**DOI:** 10.1186/s12875-020-01142-9

**Published:** 2020-04-22

**Authors:** Amy O’Donnell, Barbara Hanratty, Bernd Schulte, Eileen Kaner

**Affiliations:** 1 Population Health Sciences Institute, Newcastle University, Baddiley-Clark Buiding, Richardson Road, Newcastle, NE2 4AX UK; 2 Centre of Interdisciplinary Addiction Research (ZIS), Department of Psychiatry and Psychotherapy, University Medical Center Hamburg-Eppendorf, Hamburg, Germany

**Keywords:** Alcohol prevention, Normalisation process theory, Qualitative research, Primary health care, Patients

## Abstract

**Background:**

Despite evidence supporting the effectiveness of alcohol screening and brief advice to reduce heavy drinking, implementation in primary healthcare remains limited. The challenges that clinicians experience when delivering such interventions are well-known, but we have little understanding of the patient perspective. We used Normalization Process Theory (NPT) informed interviews to explore patients’ views on alcohol screening and brief advice in routine primary healthcare.

**Methods:**

Semi-structured qualitative interviews with 22 primary care patients who had been screened for heavy drinking and/or received brief alcohol advice were analysed thematically, informed by Normalisation Process Theory constructs (coherence, cognitive participation, collective action, reflexive monitoring).

**Results:**

We found mixed understanding of the adverse health consequences of heavy drinking, particularly longer-term risks. There was some awareness of current alcohol guidelines but these were viewed flexibly, depending on the individual drinker and drinking context. Most described alcohol screening as routine, with clinicians viewed as trustworthy and objective. Patients enacted a range of self-regulatory techniques to limit their drinking but perceived such strategies as learned through experience rather than based on clinical advice. However, most saw alcohol advice as a valuable component of preventative healthcare, especially those experiencing co-occurring health conditions.

**Conclusions:**

Despite strong acceptance of the screening role played by primary care clinicians, patients have less confidence in the effectiveness of alcohol advice. Primary care-based alcohol brief advice needs to reflect how individuals actually drink, and harness strategies that patients already commonly employ, such as self-regulation, to boost its relevance.

## Background

Primary healthcare provides an ideal context for the early detection and secondary prevention of excessive alcohol consumption due to its high population coverage [1], and the frequency with which heavy drinkers present [2, 3]. Primary care clinicians can help patients reduce their drinking by identifying individuals potentially at risk using a validated screening questionnaire [4], and providing brief advice to those needing support [5]. However despite extensive evidence for the effectiveness of alcohol screening and brief advice [6], the evidence to practice gap remains wide [7]. Few clinicians screen their patients systematically for heavy drinking, and often those identified as problem drinkers fail to receive appropriate care [8]. A recent population survey in England found that fewer than one in ten heavy drinkers (defined in the UK as consuming more than 14 standard drinks per week [9]) reported having received advice on their alcohol consumption from a primary care clinician [10].

Many factors determine the rate and scale of implementation of health interventions, including the knowledge, skills and beliefs of clinicians and patients, as well as the socio-political context in which these individuals operate [11–14]. The challenges experienced by general practitioners (GPs) in delivering alcohol screening and brief advice are well-established, including lack of training [15], time [16], and financial incentives [17], alongside a fear of upsetting patients by asking about their drinking [18]. However, we have little understanding of patients’ perspectives on either being questioned about their personal alcohol consumption (screened), or receiving brief advice to help them cut down. Previous surveys of attitudes towards alcohol and other lifestyle risk factors being raised by healthcare providers suggest that people are generally willing to discuss their drinking with clinicians [19–23]. Yet whilst qualitative research exploring patients’ views on alcohol screening and brief advice has been published, previous studies have either explored patients’ hypothetical perspectives on discussing alcohol with their healthcare provider [24–26], or focussed on the views of specific patient groups such as heavy drinkers [27] or women veterans [28]. As such, there is limited evidence of patients’ real world experiences of discussing their own alcohol consumption as part of routine consultations.

**Fig. 1:**
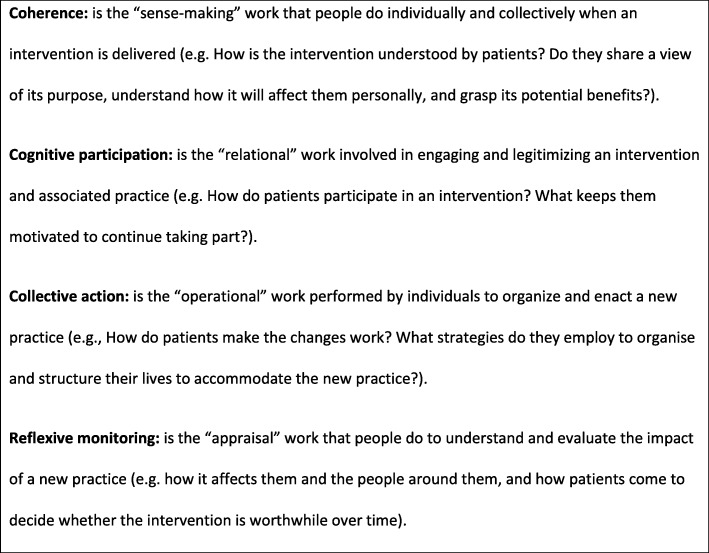
Core constructs of Normalisation Process Theory

Our qualitative study aimed to explore how patients understand and engage with either being screened for heavy drinking and / or receiving brief alcohol advice, when such practices are implemented in everyday primary care. We used Normalisation Process Theory (NPT) to inform the design, analysis and interpretation of the interviews [29]. NPT provides a robust theoretical framework to help explain the ‘work’ involved in implementing a set of healthcare practices through the operation of four mechanisms: coherence; cognitive participation; collective action; reflexive monitoring (Fig. 1).

## Methods

Seven primary care practices in Northern England participated. Patients were identified by practice administrative staff using standardised GP Read Code searches on the basis of their electronic medical record as having: (1) been asked about their alcohol consumption (number of alcohol units consumed per week recorded); (2) screened for heavy drinking using a validated questionnaire (recorded administration of either the Alcohol Use Disorder Identification Test (AUDIT), its abbreviated version (AUDIT-C), or the Fast Alcohol Screening Test (FAST) [30]); and / or (3) received brief alcohol advice from their GP or nurse in the past 6 months (recorded delivery of alcohol advice or a brief or extended alcohol intervention). Interested participants were contacted via telephone or email, at which point the confidential nature of the study was emphasized. We aimed to recruit a maximum variation sample, comprising patients with different socio-economic and demographic characteristics.

**Fig. 2:**
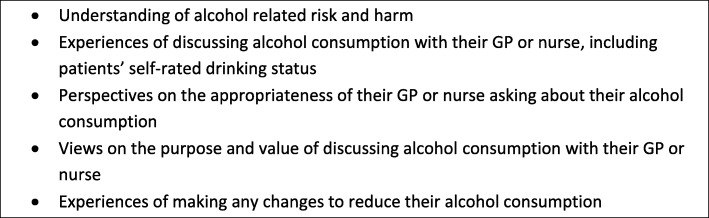
Focus topics in the patient interviews

Interviews were arranged at a convenient time and place for participants, generally their primary care practice or home. All interviewees gave written informed consent and received a £10 shopping voucher for their participation. Interviews took place between April 2016 and January 2017, lasted 18 to 83 min (mean = 39), were audio-taped, and transcribed verbatim. A topic guide focused discussion around the broad study aims (see Fig. 2), with emergent or unforeseen issues explored as relevant. Interviews were conducted until no new information or themes were emerging [31].

First, we used Framework Analysis [32] to sift, chart and sort the interview data in a five step process: (1) familiarisation; (2) identifying a thematic framework; (3) indexing; (4) charting; (5) mapping and interpretation [33]. The lead researcher (AOD) conducted initial analyses and coding, with themes agreed, refined and developed through discussion with the wider team. Next, we conducted further theory-driven data analysis, whereby the initial inductive themes were mapped against the four NPT constructs [34]. This latter stage was an iterative process, moving back and forth between and within inductive and theory-driven themes [35]. Two researchers (AOD, BSc) conducted this analysis individually, with the final analysis agreed via discussion. This two-stage approach helped to avoid “forcing” our data into predetermined conceptual categories, and thus ensured our interpretation remained data-driven [36]. NVivo Qualitative Research Software (v11: QSR International, Cambridge, MA, USA) was employed to support data management and analysis.

## Results

All 22 interviewees were white British and aged between 25 and 75 years (mean = 54). A balanced representation of gender was achieved, and participants’ were drawn from across the socio-economic spectrum, as measured by occupation [37]. Most participants self-rated their alcohol consumption as being at a lower-risk level, six indicated heavy drinking, and one identified as a recovering dependent drinker. Although all participants recalled being asked about their alcohol consumption in routine appointments (screening), only three patients reported that they had received brief advice for drinking above recommended amounts. Most participants (*n* = 11) had been registered at their practice for less than a year, reflecting current UK policy targeting alcohol screening at newly-registered patients (Table 1) [38].

**Table 1:** Characteristics of patients interviewed

ID	Gender	Age	Relationship status	Children (Yes/No)	SOC^1^	Years at practice	Self-rated drinking status	Screening experience	Received brief advice
P1	Male	70–79	Married	Yes	5	40–49	Low risk	Annual health check	No
P2	Male	60–69	Married	Yes	6	40–49	Low risk	Annual health check	No
P3	Female	60–69	Divorced	Yes	7	< 1	Hazardous	New registration	No
P4	Female	50–59	Married	Yes	6	30–39	Low risk	Annual health check	No
P5	Male	70–79	Married	Yes	1	< 1	Hazardous	New registration	Yes
P6	Female	40–49	Divorced	No	2	10–19	Former dependent	N/A	N/A
P7	Male	50–59	Married	Yes	2	1–9	Low risk	New registration	No
P8	Male	50–59	Single	No	1	< 1	Hazardous	New registration	No
P9	Male	50–59	Single	No	8	1–9	Low risk	Annual health check	Yes
P10	Male	50–59	Single	No	8	10–19	Low risk	Annual health check	No
P11	Female	60–69	Partner	Yes	6	30–39	Low risk	40+ health check	No
P12	Male	20–29	Single	Yes	2	< 1	Hazardous	New registration	No
P13	Male	50–59	Married	Yes	7	< 1	Low risk	New registration	No
P14	Female	20–29	Partner	Yes	4	< 1	Low risk	New registration	No
P15	Female	50–59	Partner	Yes	2	< 1	Low risk	New registration	No
P16	Male	40–49	Partner	Yes	5	< 1	Low risk	New registration	No
P17	Female	60–69	Partner	Yes	2	< 1	Low risk	New registration	No
P18	Male	40–49	Married	Yes	5	10–19	Low risk	40+ health check	No
P19	Female	50–59	Married	No	1.1	1–9	Hazardous	General consultation	No
P20	Female	40–49	Married	Yes	3	10–19	Hazardous	40+ health check	Yes
P21	Male	50–59	Partner	Yes	1.2	< 1	Low risk	New registration	No
P22	Female	60–69	Single	Yes	3	< 1	Low risk	New registration	No

^1^Social grade was measured using the Standard Occupational Classification (SOC), where 1 = highest and 8 = lowest.

Key themes from the interviews are summarised in Table 2, with the main body of results structured around the four NPT constructs.

**Table 2:** Summary of key themes and sub-themes by NPT construct

NPT construct	Theme	Sub-theme
Coherence	Patients’ understanding of alcohol-related risk and harm	• Knowing the limits: what it means to drink to excess
		• Awareness of immediate versus longer term risk
Cognitive Participation	Engaging with screening and brief interventions in primary care	• Alcohol conversations as standardised and routine
		• Primary care clinicians viewed as trustworthy, objective and expert
		• Telling the truth about drinking
Collective action	Managing lower risk alcohol consumption in everyday life	• Personal strategies to limit alcohol consumption
		• Influence of families and relationships
Reflexive monitoring	Impact of alcohol advice on individual drinking practices	• Value of talking to patients about alcohol
		• Challenge of dealing with heavy drinkers

### Patients’ understanding of alcohol-related risk and harm

Discussions around coherence (or sense-making) revealed a degree of dissonance between patients’ understanding of the purpose of screening and brief alcohol advice, and how alcohol-related risk was viewed in their everyday lives.

#### Knowing the limits: what it means to drink to excess

Patients gave varying descriptions of what they believed to constitute lower risk alcohol consumption. Some referred to the maximum number of units currently recommended for men and women by the UK Department of Health (14 per week). However, many thought this number should vary for different individuals, depending on their age, gender or size:

*My perception is that the bigger somebody is, or the taller, the larger...they could take more alcohol that myself, who is five foot one and eight stone. I don’t think I could drink as much alcohol as somebody who is six foot and seventeen stone. (P17, Female, 60–69 years)*

There was shared understanding that engaging in heavy episodic (‘binge’) drinking was problematic compared to spreading consumption across the course of a week. Yet for many patients, excessive drinking was less about the quantity consumed, and more to do with an individual’s emotional relationship with alcohol. As such, lower risk consumption was conceptualised as drinking in a way that felt controlled, relaxing and enjoyable. In contrast, problematic consumption was associated with ‘needing’ to drink and drinking to become ‘drunk’. Indeed, lack of control over consumption was a strong recurring theme when patients talked about what characterised problem level drinking:

*If you’re drinking a lot to the point where it’s kind of affecting you, or you’re becoming dependent on it, then I think that you’ve got a problem. I’d probably be a little bit less concerned about the actual amount. (P8, Male, 50–59 years)*

#### Awareness of immediate versus longer term risk

Few patients considered the medium to long-term health impacts of excessive alcohol consumption, although there was some awareness of the association with liver disease. The majority focussed on immediate (or acute) alcohol-related risk, such as the higher likelihood of causing accidents to others (for example by driving-under-the-influence), or being at increased personal risk whilst drunk. Combined with a perception of immediacy, alcohol-related risk was framed in spatial or social terms. For example, drinking in unfamiliar or unsafe spaces and situations was seen as high risk.

*‘Risky drinking’ would be drinking too much so that you haven’t got control of yourself or the situation and are putting yourself at risk … Say you are out in [name of city] in a bar, you have too much or more than you should and you haven’t really got control over what’s happening to you... (P19, Female, 50–59 years)*

Risk was also associated with particular drinking practices that could heighten one’s vulnerability. For example, drinking a large amount in short space of time, and consuming particular types of drinks. Risk was also viewed as being shaped by age and gender: younger people were viewed as particularly ‘risky’ drinkers as a result of pre-loading; young women were seen as more at risk when inebriated in public spaces:

*I would think it would be somebody - say, like a young girl, 18- or 19-year old - going out on a Friday and Saturday night and getting so drunk that they put themselves in a vulnerable position (P15, Female, 50–59 years)*

### Engaging with screening and brief interventions in primary care

Patients’ cognitive participation in (or engagement with) screening and brief alcohol advice required them to perform a range of relational work with primary care clinicians. However, whilst there was widespread acceptance of primary care as a legitimate setting for preventative alcohol activities, some questioned the extent to which patients were likely to engage openly in conversations about their drinking.

#### Alcohol screening as standardised and routine

Most patients recalled being asked about their alcohol consumption at one of three types of appointments: registering as a new patient; during an NHS Health Check for adults aged 40 to 75; or within the ongoing management of a long-term health condition. Few were screened outside these situations. One patient had raised the subject of alcohol in connection with other health concerns, and another described her experience of trying to access specialist support for dependent drinking via her GP. Most found the screening experience relatively straightforward, including those that had self-administered the questionnaire in the practice waiting room. Patients remembered little about the content of the screening conversation, although those that could recall specific questions suggested they focussed on asking how many units of alcohol they drank:

*If I remember rightly, it’s like, how many units, how often? Did I ever think I binged, type of thing?... it wasn’t insulting. I didn’t take offence... (P4, Female, 50–59 years)*

Only three interviewees recalled receiving any advice after screening positively for heavy drinking. One patient had screened positively after reporting infrequent heavy episodic drinking. She recalled that the nurse had suggested she would be better advised to drink smaller amounts more frequently. As an infrequent drinker, she found this confusing, as it was perceived to encourage increased alcohol consumption overall:

*She started … ‘Had anyone shown any concern over the amount I drank? Had I ever lost time because of the amount I drank? Did I struggle getting through the day without a drink?’...Well, she realised it was ridiculous asking me … and she said, ‘I’m sorry I have to ask you these; it’s just the box that has taken you straight to that box’ and I was like, okay, that’s fine... she said ‘it would have been better if you had a glass of wine a night instead of saving them all up’. That’s the healthier way to do it. (P20, Female, 40–49 years)*

The only patient referred to specialist treatment for dependent drinking had instigated the conversation herself. Other patients with long-term health conditions talked about having regular discussions with practice nurses about their drinking. However, these patients did not construe such conversations as structured alcohol-focussed advice, but rather as part of the broader support they received to manage ongoing health issues.

#### Primary care clinicians viewed as trustworthy, objective and expert

Alcohol consumption was generally viewed as a topic that patients were happy to discuss with their clinician. Compared to talking to friends or family about their drinking, participants described GPs and nurses as providing impartiality, the appropriate skills and expertise, and being able to signpost patients to specialist services if needed:

*It’s just a doctor will have his point of view based on facts and figures and general knowledge and what they’ve learnt over time. A friend only knows what they know. (P12, Male, 20–29 years)*

Most interviewees thought that GPs and nurses asked patients about alcohol because of concern for their health. Participants felt that the key issue was to ensure that such conversations were delivered in a non-judgemental way, and that clinicians had sufficient time to listen to their concerns. Some older patients had been registered at their practice for many years and had developed open and trustful relationships with their clinician, usually a nurse. Whilst no patients expressed concerned about having information on their own alcohol consumption recorded electronically on the practice computer, many thought that heavy drinkers could be less comfortable.

#### Telling the truth about drinking

When clinicians asked about their drinking, most patients suggested that they responded truthfully, even when they were aware that their consumption exceeded recommended limits. Participants articulated a belief that honesty underpinned an effective doctor-patient relationship: the clinicians’ job was to ‘keep you healthy’; the patients’ was to provide enough information to support that role. This was particularly important for patients with ongoing health conditions, as the following patient, who identified herself as a hazardous drinker, describes:

*They asked me how many units and I told them honestly. I am aware some people tend to skirt around the issue but because I was having health problems and there were problems with the blood results that I had, I was perfectly honest about the number of units that I was drinking. (P19, Female, 50–59 years)*

Further, for patients that viewed their own drinking as unproblematic, there was no compulsion to conceal any information, even if they recognised that the level of consumption exceeded official recommendations. Several patients communicated a dissonance between what health providers perceived as problematic drinking and how they viewed their own relationship with alcohol. In particular, those that had screened positively as a result of reporting heavy drinking connected with a social event were reluctant to have their consumption labelled as ‘excessive’. One patient recalled such an instance:

*I said, ‘I’ve just been home for a weekend with the lads, the first one we’ve had for a long time.’ I said, ‘We went to the races.’ And he said, ‘Did you drink?’ I said, ‘Oh, yes.’ I said, ‘I had quite a good day, I had a meal...We had a good night … I drunk nine pints of beer.’ That’s nothing really at the time, I mean. And he put down on my report, ‘Alcohol Abuse.’ … I said, ‘Nine pints? Over about a six or seven-week period? I don’t think so.’ (P2, Male, 60–69 years)*

However, there was a strong belief that individuals who privately identified as a heavy drinker could be less likely to tell the truth (*… if I did drink excessively … I could see why that would be something that would make you uncomfortable”. P14, Female, 20–29 years)*. Indeed, a few patients described how they deliberately shaped responses to questions about alcohol consumption to reduce the chance of being flagged as having a problem, for example:

*“I did put less than what it would have been … because I know if I’m going to put it down truthfully they are going to come back to me saying, ‘You have got a problem’.” (P12, Male, 20–29 years)*

### Managing lower risk alcohol consumption in everyday life

When considering the operational work undertaken to make positive changes in their drinking behaviour (collective action), few patients identified any advice obtained from their GP or nurse as influential. Rather, alongside various social or familial influencers, patients described a range of self-regulatory practices they enacted to limit alcohol consumption in daily life.

#### Personal strategies to limit alcohol consumption

Many positioned alcohol as a ‘treat’ that should be reserved for special and social occasions. Those patients that saw themselves as lower risk drinkers also emphasised how they remained in control of their drinking by self-monitoring their alcohol intake. As part of this approach, it was important to ensure that alcohol was less accessible, such as by limiting availability at home. Participants also evidenced control by describing situations in which they had turned down an alcoholic drink or substituted it for a soft or lower strength beverage:

*If you like drinking beer, the weaker it is, the more you can drink...I look at the bottle and say, “That’s 500ml and its 3.8, so that’s 1.9 units ticked off. I can’t have too much more. (P5, Male, 70–79 years)*

Some patients viewed limiting their alcohol intake as an intrinsic component of a healthy lifestyle. Female participants in particular mentioned concerns about weight-gain as influencing lower consumption. However, several patients believed that it was possible to mitigate the worst effects of excessive alcohol consumption by adopting other positive lifestyle behaviours, such as eating well or exercising regularly. Heavy drinking could be balanced out in this way, either on a day-to-day basis, or as part of a cycle of binge-then-cleanse.

#### Families and relationships

Friendships, families and relationships were identified as influencing drinking practices in multiple ways. Parental responsibilities were mostly seen as encouraging reduced consumption. This resulted from both immediate practical concerns, such as needing to be sober to be able to parent younger children effectively, to more long-term parenting ambitions, such as wanting to be a positive role model. A few participants who recounted difficult experiences due to having a close family member dependent on alcohol discussed how this had made them less likely to drink excessively themselves.

Drinking practices established within intimate relationships were also seen as influential. If one partner made a conscious decision to reduce their drinking, this could impact positively on the other. However, relationship breakdown was identified as a prime trigger for increased alcohol consumption. An alcohol dependent participant described a history of traumatic experiences, including multiple instances of bereavement, difficult relationships and financial stress. She identified links between these critical life events and points at which her level of drinking escalated, and/or sense of control over her drinking became substantially impaired.

*I’d lost my mum in 2004. And then, on the day that my mum’s funeral was, I discovered that my bookkeeper … had stolen £250,000 from one of the businesses … Really, my alcohol intake had escalated from that point. (P6, Female, 40–49 years)*

### Impact of alcohol advice on individual drinking practices

Considering their experiences of preventative alcohol discussions in primary care (reflexive monitoring), there was a general perception that it was a worthwhile endeavour for patients. However, the impact of alcohol advice was contingent on whether an individual was sufficiently motivated to change their drinking practices, particularly when such practices were socially normalised.

#### Value of talking to patients about their drinking

Patients with long-term health conditions said they found it helpful to talk about their drinking with practice nurses. There was a perception that regularly discussing alcohol consumption alongside other lifestyle behaviours had a beneficial impact on the ongoing management of their specific condition, as well as their wider health and well-being. Yet irrespective of health status, most believed it was important that clinicians asked about alcohol in routine consultations. This was mainly because alcohol consumption was viewed as a core component of standard primary care health advice:

*They go on about people having diabetes, having heart problems, and your blood pressure, and all of these things seem to be very important as you get older. I think if people can either take the information and use it or just ignore it. (P11, Female, 60–69 years)*

Although patients recognised that such advice might not always be immediately impactful, it was nevertheless important to provide individuals with the information they needed to make healthier lifestyle choices as the conversation could plant a seed for future contemplation, which could eventually trigger positive change:

*It might just open a door, a little chink of light to the one filling in the form, or to the doctor, just a signal that there’s something happening. (P21, Male, 50–59 years)*

#### Challenge of dealing with heavy drinkers

Whilst asking about an individual’s alcohol consumption was viewed as important, many questioned the extent to which heavy drinkers would actually be able to change their behaviour based on simple brief advice. For some, this related to the limited time that primary care practitioners have to spend with their patients. Others felt that peer support groups needed to be involved in the provision of interventions for heavy drinking. Current or former heavy drinkers talked about their reluctance to discuss alcohol with their GP due to the stigmatised nature of dependency, limited belief in their ability to control consumption, and occasionally, because they recognised they were not ready or willing to change:

*When they say, ‘If you drink more than 20 units a week,’ you don’t care … It doesn’t make a ha’p’orth* [half-pennyworth] *of difference to an alcoholic, or an alcoholic-dependent person, or an addictive personality. It doesn’t. (P6, Female, 40–49 years)*

## Discussion

Most patients reported finding it acceptable for clinicians to ask about their alcohol consumption (cognitive participation), and viewed such work as valuable component of preventative healthcare, particularly those experiencing co-occurring health conditions (reflexive monitoring). At the same time, participants suggested that ‘others’, particularly heavy drinkers, might feel less comfortable about a clinician raising the topic of alcohol, and might be less inclined to tell the truth about their drinking. Previous studies have highlighted how this process of ‘othering’ can skew perceptions of personal risk, making it less likely an individual will recognise their own need to reduce consumption [39, 40]. A complex picture emerged around patients’ understanding of the purpose of alcohol screening and brief advice (coherence), with their awareness of the adverse health consequences of heavy drinking often outweighed by the pleasurable experiences and positive emotions associated with alcohol consumption.

Whilst patients enacted a range of strategies to limit their drinking (collective action), there was mixed evidence as to whether primary care advice influenced subsequent alcohol consumption. Two possible reasons were identified. First, despite reasonable awareness of current UK drinking guidelines, patients were sceptical of the extent to which these limits should be applied universally. As such, whilst brief advice might be appropriate for ‘other’ drinkers, it was often construed as irrelevant to the individual concerned. Second, whilst supportive in principle for the delivery of brief advice by clinicians, in reality patients were sceptical about its potential to affect their drinking. Instead, participants described a range of experientially-derived rules and practices they employed to help control consumption in everyday life.

A key strength of our study is the use of a robust middle-range theory (NPT) as an analytical lens through which to explore patient’s experiences of alcohol-related conversations. As others have found, using NPT encouraged us to broaden themes of enquiry beyond the usual barriers and facilitators considered in this field to develop more a nuanced and insightful understanding of the patient experience [35]. For example, as well as encouraging a focus on patients’ ‘coherence’ of alcohol screening and brief advice, it also allowed us to unpick and reflect on the various layers of relational ‘work’ they are implicitly expected to carry out when engaging with alcohol screening and brief advice in primary healthcare.

At the same time, whilst we employed a two-stage approach to analysis, involving an initial inductive thematic approach, we acknowledge that using NPT to develop sensitising questions may have influenced emergent themes. Further, although we endeavoured to develop study-specific meanings of each NPT construct, it was sometimes challenging to apply the relevant theoretical concepts to our data. Most participants were able to reflect on their personal experiences of being asked about their alcohol consumption (screening), and could provide general perspectives on the value, relevance and acceptability of primary care-based interventions. Yet few patients could describe details of any advice they had received to reduce their alcohol consumption, and thus reflect on the impact of such advice on their drinking behaviour.

The fact that only three of the six patients who identified themselves as hazardous drinkers were able to recall receiving any alcohol advice from their GP or nurse reflects the evidence to practice gap highlighted earlier [10, 41]. We were not able to verify which patients had actually received alcohol advice, meaning it is possible that more participants had received alcohol advice but did not recall it (particularly as advice giving may have occurred up to six months before the interview took place). However, other studies suggest that patients might struggle to recollect these conversations because the advice provided in primary care is confused and ambiguous [42, 43]. As such, our findings build on those from previous implementation research to highlight the need for improved GP and nurse training to increase both the rate and quality of alcohol advice provision [44].

Existing literature also supports our conclusion that most patients find it acceptable for a GP or nurse to ask about their drinking, and support the delivery of brief advice to those drinking above recommended levels [24–27]. Yet as others have also found, there is a perception that heavier drinkers (‘others’) are more likely to be defensive to statements about the health risks associated with drinking [45], less inclined to tell the truth about their alcohol consumption [46], and less supportive for routinely asking patients about their alcohol intake than those drinking at lower-risk levels [19]. In contrast to earlier studies, however, by interviewing patients about their real-world experiences of alcohol conversations, our findings arguably have added validity. We acknowledge, however, that our sample included a relatively small number of hazardous drinkers, and even fewer participants that could recall receiving brief alcohol advice. It is possible that selection bias could have affected recruitment, meaning that heavy drinkers were less inclined to participate in an interview than those consuming alcohol at lower risk levels. Again, however, this could also result from the low levels of alcohol advice giving in routine primary care, as well as reflecting the prevalence of hazardous drinking in the general patient population [47].

Our study also supports previous evidence showing that whilst patients are aware of current UK guidelines [48, 49], real-world perceptions of ‘how much is too much’ often diverge from epidemiological constructs of risk [27, 28, 50–52]. As such, we further highlight how lifestyle behaviours, and the knowledge, attitudes and beliefs that inform such activities, are heavily socially-situated [53]. Friendships, and romantic and familial relationships exert significant influence [54], with common lifestyle practices and beliefs tending to cluster across social networks [55, 56]. Individual interpretations of risk are built on instances of death and illness that individuals have previously discussed, observed or experienced; a concept known as ‘lay epidemiology’ [57]. Lovatt et al. have employed this concept to help explain the dissonance that occurs between alcohol guidelines based on traditional epidemiological evidence, and how individuals apply such guidelines to their own drinking practices [58]. We echo their recommendation that to boost patients’ coherence, GPs need to articulate messages around lower risk alcohol-consumption in a manner which embraces the social concerns and cultural values that actually shape day-to-day drinking practices.

## Conclusion

Our findings demonstrate general support for the delivery of screening and brief alcohol advice in primary care. Thus, despite GPs’ concerns that alcohol is a sensitive topic to raise in consultations [18], they should be reassured that for many patients, such conversations are unlikely to cause offence [59]. At the same time, it is clear that messages about alcohol-related risk are failing to filter through to patients in terms of changing views or relevant health behaviour. Public investment in well-designed social marketing campaigns could help boost public awareness of, for example, the link between alcohol and cancer [60]. However, our study also suggests a need to improve existing training and support materials for primary care clinicians to enhance the relevance and potential impact of the alcohol brief advice they provide. First, by encouraging clinicians to contextualise conversations about alcohol to consider ‘how’ and ‘why’ patients actually drink rather than simply ‘how much’. By exploring the ‘bundle of practices’ that might shape situations in which excessive alcohol consumption is more likely to occur, clinicians could help patients identify more relevant strategies to reduce their drinking without incurring significant social or personal disruption [59, 61–64]. Second, by ensuring that the lifestyle advice delivered to heavy drinkers incorporates or reinforces more explicitly the self-regulating drinking strategies that individuals already enact in daily life. Many patients articulated a commitment to self-regulate their alcohol consumption and to actively limit home drinking. Recognising, encouraging and developing these positive behaviours could lend greater credibility and meaning to alcohol-related discussions in healthcare, and strengthen individuals’ capacity to shape their own health [65].

## Data Availability

Interview transcripts cannot be made available because they contain information that could compromise participant privacy which would violate the principles of the ethics committee approval.

## Abbreviations

AUDIT: Alcohol Use Disorder Identification Test
AUDIT-C: Alcohol Use Disorder Identification Test – Consumption
FAST: Fast Alcohol Screening Test
GP: General Practitioner
NPT: Normalisation Process Theory
